# Prevalence of and socioeconomic gradient in low birth weight in Ethiopia: further analysis of the 2016 demographic and health survey data

**DOI:** 10.1186/s12884-020-03313-z

**Published:** 2020-10-08

**Authors:** Gebretsadik Shibre, Mulugeta Tamire

**Affiliations:** 1 Department of Reproductive, Family and Population Health, School of Public Health, Addis Ababa University, Addis Ababa, Ethiopia; 2 Department of Preventive Medicine, School of Public Health, Addis Ababa University, Addis Ababa, Ethiopia

**Keywords:** Socioeconomic position, Low birth weight, Ethiopia demographic and health survey, Ethiopia, Socioeconomic gradient, Global Health

## Abstract

**Background:**

Evidence suggests appearance of socioeconomic gradient in the probability of low birth weight (LBW). Such evidence, however, is scanty in Ethiopia. The study aimed to examine the prevalence of and socioeconomic gradient in LBW in Ethiopia.

**Method:**

Data for the study were drawn from the Ethiopia Demographic and Health Survey conducted in 2016. The 2016 EDHS is the fourth wave in the series of nationally representative household surveys carried out in the country to deliver up-to-date health and demographic indicators for the Ethiopian population. Women aged 15 to 49 years were the main focus of the survey, with data also gathered from men aged 15 to 59 years and under five children. The study pursued complex sampling strategy to draw samples representative at national as well as at urban and rural levels. The data are available to the public domain and were accessed from the MEASURE DHS following registration. Multivariable logistic regression model and marginal standardization were used to estimate socioeconomic gradient in the probability of LBW. We performed sensitivity analysis to evaluate variation of LBW according to different categories of socioeconomic position. Maternal education and household wealth were used as measures of the socioeconomic position in the study.

**Results:**

13.2% (95% confidence interval = 10.73, 15.65) of births were complicated by LBW. The findings showed that socioeconomic gradient was evident between maternal education and LBW; as education increases from no education to secondary education, the probability of occurrence of LBW consistently declined. However, no gradient in LBW was detected for household wealth.

**Conclusions:**

We have identified education gradient in LBW, with the highest burden of LBW occurring among the non-educated women. To redress the observed education disparity in LBW, targeted interventions need to be implemented with greater emphasis placed on illiterate women.

## Background

An estimated 15 million babies are born preterm (born before 37 completed weeks of gestation) annually worldwide [1]. Globally, prematurity related complications remain the leading cause of under-five mortality [2]. Not only does prematurity lead to mortality, but it has also been shown to be a driver of many health problems such as developmental delay and Low Birth Weight (LBW) [3, 4].

According to the World Health organization (WHO), LBW is defined as weight at birth of less than 2500 g [5]. Of all births globally, 15 to 20% are estimated to have LBW, which would translate into over 20 million births annually [6]. According to the lancet report in 2019, the 2015 LBW prevalence varied 14 to 17% globally, with more than 90% occurring in low-and-middle income countries. Southern Asia and sub-Saharan Africa are home to a staggering 48 and 24% of the global LBW burden, respectively [7]. Between 2000 and 2015, no region globally decreased low birth weight prevalence significantly. Evidence has shown that prevalence of LBW had been reduced by 1.2% each year between 2000 and 2015 worldwide [8], suggesting insufficient progress required to attain the 2025 World Health Assembly low birth weight target of 30% [9]. In terms of country level distribution of low birth weight in the region of SSA, there are some within country variations [10]. The burden of LBW in Ethiopia is nearly half of the SSA average at around 11% [11].

LBW remains a substantial public health challenge worldwide [6] as it amounts to a higher risk of mortalities and morbidities for neonates and infants. Literature suggests that infants born with low weight are 25 times more likely to die compared with their counterparts who weigh 2500 g or more at birth [12]. Further, infants born with low weight suffers long term consequences such as poor school performance [13], cognitive dysfunction and low intelligence quotient (IQ) [14, 15]. LBW has also been found to substantially elevate risks of obesity and non-communicable diseases such as diabetes and cardio-vascular problems [16, 17]. A devastating nature of LBW is that its negative health consequences continue into later in life and seriously impair the normal functioning of an individual. In general, LBW comes with a myriad of social and economic burdens for a country [18, 19] and needs to be a priority nationally as well as worldwide. Mothers need appropriate nutrition, enough rest, acceptable maternal health service such as antenatal care attendance, and a clean environment to raise a healthy baby [8]. This is because, these elements would help in the recognition, prevention and treatment of problems that would potentially lead to low birth weight, and this could eventually be translated into achievement of the World Health Assembly nutrition target to reduce low birth weight by 30% between 2012 and 2025 [9].

Despite LBW being a pervasive problem worldwide, highest prevalence is reported in the poverty stricken areas of the world, Sub-Saharan Africa and Southern Asia [7], suggesting its strongest link with socioeconomic position, or Socio-Economic Status (SES) [20, 21]. For this reason, understanding the nature of relationship between LBW and SES is helpful to launch a policy to help battle the problem more efficiently in resource-strapped settings like Ethiopia. Under the ambitious Sustainable Development Goals (SDG) [22], countries have pledged to achieve health goals with “no one left behind”. The ethical marker “no one left behind” urges countries to ensuring equity, where everybody, irrespective of their SES and other grounds, should get access to health care services equitably. SES driven disparity in LBW would translate to the fact that, women at the poorer end of wealth status are at increased risk of delivering LBW babies [23] though an improvement in SES might not necessarily result in more returns in terms of reduction of LBW. Indeed, studies have shown the presence of a curvilinear relationship between SES and LBW [24] where improvement in SES does not correspond to lower rate of LBW after a certain limit of the SES. Studies have investigated the socioeconomic gradient in LBW in other countries [23, 24]. However, there is a dearth of evidence derived from methodologically rigorous studies on whether socioeconomic gradient exists in LBW in Ethiopia. Some literatures have looked at the relation between SES and LBW [25–28], but without looking into whether the observed association has shown gradients along the continuum of the SES. Further, all of these studies covered small geographical locations in the country without showing how LBW was related with SES nationally. Differentiating between graded and non-graded association between SES and LBW is important from the viewpoint of policy interventions; different policy interventions are required for socioeconomic inequality of LBW with and without graded association [24]. The present study thus aimed to show whether a socioeconomic gradient appears in the probability of occurrence of LBW using data extracted from the fourth and latest wave of the Ethiopia Demographic and Health Survey.

## Methods

### Data source

This paper has been prepared using data derived from the most recent wave of the Ethiopia Demographic and Health survey conducted in 2016 (2016 EDHS). The 2016 EDHS is the fourth round in the series of nationally representative cross-sectional surveys after the 2000, 2005 and 2011 EDHSs. The dataset were accessed from the MEASURE DHS and children’s file has been used for this study. Implementation of the survey was carried out under the auspices of the Central Statistical Agency (CSA) of Ethiopia, with ICF provided technical aid. The United States Agency for International Development (USAID) and other international organizations delivered financial support for the survey. The survey covered all nine regions and two city administrations in the country to produce data representative nationally as well as for urban and rural settings and nine subnational regions and two city administrations. Conduct of the survey was motivated by the need to provide decision makers with up-to-date evidence on various public health indicators such as child morbidity and mortality, maternal health services, fertility preference and domestic violence, to mention just a few.

The sampling procedure and methodology followed in the survey is available elsewhere [29]. The survey followed a stratified two stage cluster design to draw samples. The territory of Ethiopia was divided into nine subnational regions and two city administration. Stratification was conducted such that every subnational region would have urban and rural strata, except Addis Ababa which is entirely urban, followed by the selection of Enumeration Areas, EA (or clusters or primary sampling units) from each stratum independently with Probability Proportional to the Size (PPS) of that EA. This constituted the first stage of sampling. The Population and Housing Census (PHC) of Ethiopia carried out in 2007 delivered sampling frame for the choice of EAs in the first stage. Overall, 645 EAs (202 urban and 443 rural areas) were selected out of the 84, 915 EAs overall in Ethiopia. Complete household listing was accomplished in the selected EAs prior to the commencement of household selection. Then, a predetermined number of 28 households were drawn from each EA through equal probability systematic approach in the second stage of the sampling process. The survey included 5232 households in urban areas and 11,418 households in rural areas, yielding a total of 16,650 households. Eligibility for the interview included being women aged 15–49 years and men aged 15–59 years, irrespective of their residency type (permanent or visitors). In total, 16,583 women aged 15–49 years were found in all the interviewed households, of which 15,683 women were available for interview, yielding a response rate of 95%.

The 2016 survey used five questionnaires to capture information on a wide range of health issues: household, women’s, men’s, biomarker and health facility questionnaire. Since women in the reproductive age group remain the major focus of the survey, women’s questionnaire was the principal source of data and was applied to gather data on issues specific to women and their children such as maternity care, family planning, infant feeding practices, vaccinations, childhood illness and low birth weight. The questionnaires were adapted from standard DHS questionnaires to better reflect the contexts and requirements of the country.

### Study variables

The outcome variable for this study is LBW. In the survey, birth weight information was collected for children who were born 5 years prior to the interview date. In total, there were 10,641 births born 5 years preceding the survey. However, birth weight information was available for 2110 births only, comprising nearly 14% (weighted) of the sampled births in the survey. In the questionnaire used to collect the DHS data, women were asked two questions that help to collect information on LBW: 1) was (NAME) weighed at birth? and 2) How much did (NAME) weigh?. For the first question, she replied either “yes” or “no”. Women who replied “yes” were then asked to answer the second question, where there are three choices about birth weight information: weight from medical record, from mothers recall and “do not know”. So, in this study, information on birth weight was based on either written records from medical charts or recall of the mother. LBW was used as a binary variable where 1 represents children born low weight at birth (less than 2500 g) and 0 if the child weigh 2500 g or more. Multiple pregnancies have not been an issue for the study since about 97% of the babies sampled and analyzed in the study were singleton**.**

Wealth index and educational status of the mother were used as the SES measures for this analysis. Wealth index is a composite summary measure intended to reflect the economic rank of a household and is produced using a statistical procedure called Principal Component Analysis (PCA) [30, 31]. It is computed based on an approach suggested by Filmer and Pritchett [32] using household possessions such as radio, telephone and television and characteristics such as water supply and sanitation facilities [30]. Once scores for each household and participants is generated by PCA, five quintiles are formed by regrouping the scores: poorest, poorer, middle, rich and richest. Wealth index variable already computed by DHS was used for the study. The educational status of the mother was recorded as no education, primary, secondary or higher.

To measure the net influence on LBW of the socioeconomic variables, we controlled for the influence on LBW of other factors in our statistical model. Such other factors are known as confounding factors. A confounding variable associates with both a cause (SES in this study) and response variable (LBW in this study). The choice of the potential confounding variables for our study was informed by literature. Mother’s age at birth of child, ethnicity, and maternal occupation [24, 33, 34] were found to potentially confound the association between LBW and socioeconomic position. Age of the mother at birth was coded as < 20 years, 20–34 years and 35 years or older [23]. Ethnicity of the mother was classified as Amhara, Oromo, Tigrie and others. Maternal occupation was coded as not working, professional, clerical, sales, employed, services, skilled manual, unskilled manual and others. Wealth status can confound the education-LBW association and education can confound the wealth-LBW association [23].

### Statistical analysis

We examined the socioeconomic gradient involved in LBW using regression model. We run multivariable logistic regression to produce results that are statistically significant net of effect of confounding variables discussed above. Statistical significance was measured through Odds Ratio (OR), 95% confidence interval (CI) and *P*-value < 0.05. Further, marginal standardization was performed to find out predictive probability of LBW across the entire subgroups of the SES. It has been shown that this approach is suitable method to reliably estimate predictive probabilities after fitting cofounder adjusted logistic regression [35]. The SES gradient in LBW was checked through both the OR and probability curves. The marginal standardization procedure was conducted using ‘margins’ STATA module and this allowed us to carry out sensitivity analysis, allowing the estimation of probability of LBW at different values of wealth quintiles and maternal education. When a survey followed complex sampling structure and samples were drawn from stratified multistage sampling procedure, analysis of the data requires special attention to get unbiased findings as well as results representative of the intended population. The reason being, complex sampling structure results in respondents being sampled with unequal probability of selection and this in turn leads to some groups to be oversampled and others under sampled [36]. Since data in the 2016 EDHS were collected through such complex sampling method, we weighted our analysis through a weighting variable already available in the dataset to correct the problem introduced because of disproportionate sampling. We used the ‘svyset’ STATA module for this purpose. The analyses were undertaken in STATA v.13.

## Results

A total of 2110 live births in the 5 years before the survey were included in the analysis**.** Table 1 displays the various characteristics of the surveyed women. More than half of the studied women fell in the richest category of wealth quintiles. Most of the participants either did not attend formal education (28%) or completed primary schooling (38%). About half of the respondents were either Amhara or Oromo (53%). More than three-fourths of births were among women aged 20 to 34 years. The largest proportion (45%) of women did not have occupation at the time of the survey administration.

**Table 1:** Characteristic of the sampled women, 2016 (*N* = 2110)

Characteristics	Categories	Weighted percentage (%)
**Wealth index**	Poorest	6.9
	Poorer	10.3
	Middle	13.9
	Rich	16.1
	Richest	52.8
**Education of the mother**	No education	28.9
	Primary	38
	Secondary	17.8
	Higher	15.3
**Ethnicity**	Amhara	25.3
	Oromo	27.8
	Tigrie	15.4
	Others	31.5
**Age at giving birth**	14–19	10.5
	20–34	78
	> = 35	11.5
**Maternal occupation**	Not working	44.9
	Professional	7.9
	Clerical	1.9
	Sales	21.9
	Agriculture-employee	11.2
	Services	3.9
	Skilled/unskilled manual	4.9
	Others	3.3

Table 2 shows weighted percentages of LBW among different characteristics of the studied women. Overall, the prevalence of LBW was 13.2%; 95% CI = 10.73, 15.65. There was substantial amount of overlap in the 95% CI around the estimated point estimates of LBW across wealth, education and other characteristics, and makes it difficult to conclude on whether percentages of LBW differed across the categories significantly. However, based on the point estimates alone, LBW appeared to be smallest among the richest wealth quintile and highest among women in the poorer subgroup of wealth.

**Table 2:** Low Birth Weight disaggregated by the sample characteristics, 2016 EDHS (*N* = 2110)

Characteristics	Categories	Weighted percentage (%)
*Total low birth weight percentage*		13.2 (10.73, 15.65)
**Wealth index**	Poorest	11.3 (5.12, 23.3)
	Poorer	18.7 (11.44, 29.1)
	Middle	17.3 (10.9, 26.5)
	Rich	15.7 (10, 23.8)
	Richest	10.5 (7.9, 13.9)
**Education of the mother**	No education	18.3 (13.5, 24.3)
	Primary	11 (8.02, 14.9)
	Secondary	7.7 (4.9, 12)
	Higher	15.4 (9.6, 23.8)
**Ethnicity**	Amhara	15.8 (10.74, 22.6)
	Oromo	16.3 (11.28, 23)
	Tigrie	6.5 (4.32, 9.8)
	Others	11.6 (8.73, 15.31)
**Age at giving birth**	14–19	19.5 (12.1, 30)
	20–34	12.6 (10.2, 15.5)
	> = 35	11.6 (5.8, 22)
**Maternal occupation**	Not working	13.3 (10, 17.3)
	Professional	10.3 (3.5, 27)
	Clerical	2 (0.3, 13.6)
	Sales	11 (6.8, 17)
	Agriculture-employee	20.3 (13.6, 29)
	Services	5.5 (2.3, 12.8)
	Skilled manual	28.2 (12.2, 52.6)
	Unskilled manual	10.8 (3.6, 28.2)
	Others	8.2 (3.2, 19.3)

**Fig. 1:**
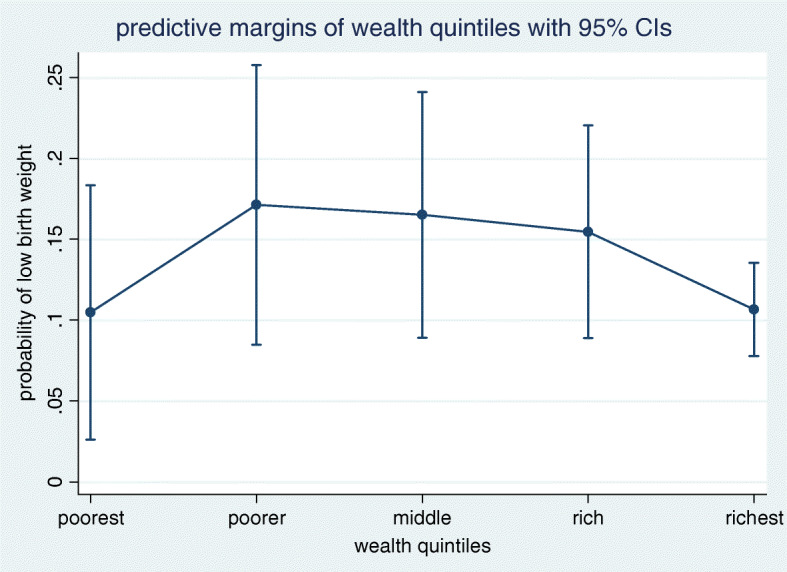
Predictive probability of low birth weight in different categories of wealth quintiles

**Fig. 2:**
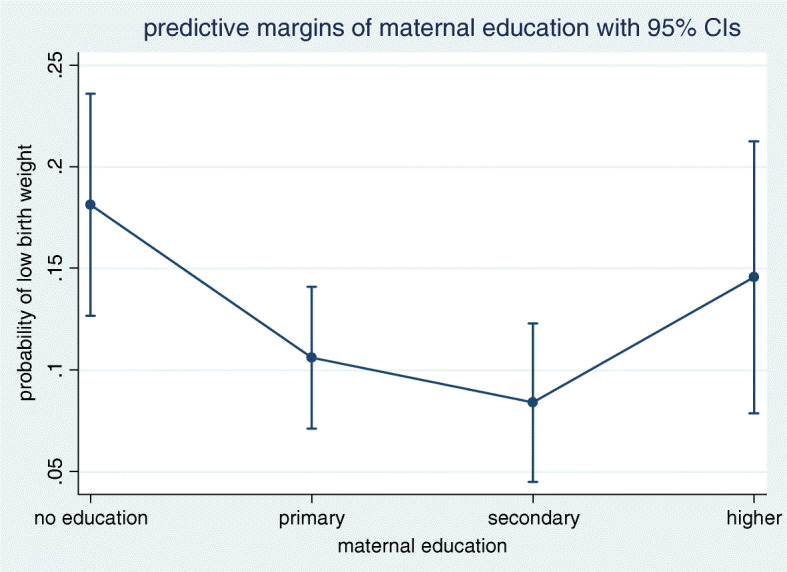
Predictive probability of low birth weight in different categories of maternal education

Table 3 portrays the result of confounder adjusted logistic regression analysis. When the effect of confounders was adjusted, the occurrence of LBW did not differ by wealth quintiles (*p*-value> 0.05 for each of the four categories compared to the poorest category). This is supported by the probability curve depicted in Fig. 1 where the 95% CI for all categories of wealth quintile overlap. However, there appeared to exist graded association between the odds of LBW and the first three subgroups of maternal education (no education, primary and secondary). For instance, the odds of giving birth to a LBW baby among secondary complete mothers fell by 63% compared with that of mothers in the ‘no education’ category(*p*-value = 0.005). This finding is also evident in the probability curve and results shown in Fig. 2 and Table 4.

**Table 3:** Odds of low birth weight by wealth index and maternal education level, 2016 EDHS (*N* = 2110)

Characteristics	Categories	AOR(95%CI)	***P***-value
**Wealth quintiles**	Poorest (ref)		
	Poorer	1.73 (0.6, 4.9)	0.302
	Middle	1.6 (0.6, 4.4)	0.359
	Rich	1.4 (0.5, 3.97)	0.496
	Richest	0.95 (0.38, 2.4)	0.917
**Maternal education**	No education (ref)		
	Primary	0.52 (0.297, 0.91)*	0.022*
	Secondary	0.37 (0.19, 0.739)*	0.005*
	Higher	0.67 (0.33, 1.352)	0.262

Note.*CI* Confidence Interval; *AOR* Adjusted Odds Ratio; *ref.* reference

*indicates significant association with LBW at 0.05 *p*-value

**Table 4:** Predicted probability of low birth weight by wealth quintiles and maternal education, 2016 EDHS (*N* = 2110)

Characteristics	Categories	Predicted probability	***P***-value
**Wealth quintiles**	Poorest	0.105	0.009
	Poorer	0.17	< 0.001
	Middle	0.165	< 0.001
	Rich	0.155	< 0.001
	Richest	0.107	< 0.001
**Maternal education**	No education	0.18	< 0.001
	Primary	0.106	< 0.001
	Secondary	0.084	< 0.001
	Higher	0.145	< 0.001

Holding all variables in the logistic regression model at their observed values, the probability of delivering a LBW baby was lowest (10.5%) among mothers in the poorest quintile (*p*-value = 0.009) and highest (17%) among ‘poorer’ mothers (*p*-value< 0.001). The probability of having a LBW baby was highest (18%) among mothers with no history of formal education, followed by 10.6 and 8.4% respectively among primary and secondary complete mothers, resulted in graded association until secondary education, as indicated by the steady decline in LBW probability as we move down from no education to secondary education. However, we did not find wealth gradient in LBW. See Table 4 for detail.

Table 5 portrays outputs of the sensitivity analysis. Our sensitivity analysis showed that the probability of LBW did not change by wealth quintiles. Making the ‘poorest’ as the reference group, we calculated percentage points (pp) by which LBW probability increased or decreased as we move from one category to others. For example, if mothers who were initially in the poorest quintile moved to the ‘poorer’, then this change caused the probability of LBW to increase by 7 pp and was not significant statistically (*p*-value> 0.05). In support of findings obtained from the regression model above, we found significant pp changes in LBW probability in relation to maternal education. If all mothers who currently fell into the ‘no education’ group moved to primary education group, the probability of giving birth to LBW baby decreased by 7.5 pp (*p*-value = 0.026).

**Table 5:** Marginal effects on the probability of low birth weight due to a change in values of wealth quintiles and maternal education, 2016 EDHS (*N* = 2110)

Characteristics	Categories	Change in probability of LBW	***P***-value
**Wealth quintiles**	Poorest (ref)		
	Poorer	0.07	0.250
	Middle	0.06	0.262
	Rich	0.05	0.347
	Richest	0.002	0.967
**Maternal education**	No education (ref)		
	Primary	−0.075	0.026*
	Secondary	−0.097	0.007*
	Higher	−0.036	0.429

Note: *ref*. reference; *LBW* low birth weight

*shows significant association at *p*-value 0.05

## Discussion

The study attempted to investigate the socioeconomic gradient involved in the occurrence of probability of LBW using the nationally representative data extracted from the 2016 EDHS. We showed that, one in approximately eight babies was born with LBW, making it one of the major public health problems to deal with.

Given its prominent role in morbidity and mortality of children, LBW remains a big obstacle to attain the neonatal mortality rate target set out for the 2030 international goals [22]. From the 2011 [11] level, LBW increased steadily, suggesting insufficient response from the concerned health bodies. But compared with a study by Assefa N et al. (2012) [28] in Oromia region, the national burden of LBW seemed to be by far lower. Apart from being confined to a more limited area, this study used data extracted from a Demographic Surveillance System (DSS) where the authors easily access objectively recorded birth weight data and thus the study is less likely to suffer misclassification of birth weights. In the current analyses of the EDHS data, information on birth weight came partly from birth card and partly from mother’s recall with the latter potentially leading to misclassification of birth weights. Due to this reason, active birth weight recording systems such as DSS can be a partial remedy in settings where vital registration system is not available. Other small scale studies conducted in different parts of Ethiopia generally found LBW that is higher than the national estimate presented in this paper [25–28]. Interestingly, LBW in Ethiopia is below half of the SSA average [7], and some between country variations were reported.

The WHO has called the member states to slash LBW prevalence by 30% by 2025 [9]. Accordingly, countries are required to reduce LBW nearly 3% annually between 2012 and 2025. This would correspond to decreasing LBW from roughly 20 million to about 14 million. However, given the increasing trends of the problem over the last five to 6 years, it is less likely for Ethiopia to attain the target of about 9% by the end of the deadline unless much attention is directed towards it. With LBW and prematurity already among the largest contributors to neonatal death worldwide, neonatal mortality related SDG would remain in peril unless countries are able to substantially cut the currently high rate of LBW.

Interesting findings emerge with respect to social gradients of LBW. The study revealed maternal education gradient in LBW in the confounder adjusted regression model. The odds of LBW were highest among illiterate, followed by ‘primary’ and ‘secondary’ education subgroups. The odds of LBW among mothers who completed primary and secondary education, respectively, were nearly 50 and 60% lower than among women without formal education, suggesting pattern of increasing returns (in terms of decreasing LBW) until secondary education, after which no reduction in LBW was observed. The pattern we found in the present study looks roughly like curvilinear graph typically found in studies involving SES gradient for health where a decreasing health gain was observed after a certain SES threshold [24]. Although the exact shape of the gradient in LBW in Ethiopia needs to be confirmed by future studies, we discovered here that increasing maternal education up until secondary education can significantly reduce the odds of LBW; after which point no association was discovered. The current finding is supported by existing evidence that maternal education has exhibited a graded relationship with LBW in United Kingdome but not in America, Canada and Australia [23] though the two studies differed methodologically.

Our sensitivity analysis on maternal education gradient for LBW supported the result we obtained from the regression analysis. The probability of occurrence of LBW varies with subcategory of mother’s education. Holding illiterate women as a reference category, the probability of LBW significantly dropped as we move down to the ‘primary’ education level from the ‘illiterate’ subgroup. Similarly, an even significantly higher drop in LBW was observed as the educational status of the mother changes from ‘no education’ to ‘secondary’ education (Table 5). This gradual decline in the prevalence of LBW down the sub-categories of maternal education evidently tells the responsiveness of LBW; the higher the maternal education (up to secondary level), the less probability of LBW.

However, given substantial inconsistencies in the literature around this issue, more context specific studies are still needed to provide conclusive evidence on the nature of relationship between SES and LBW. In contrast to findings elsewhere [23, 24, 28], we did not observe association between wealth index and LBW. In fact, our analysis approach differed from that of the prior studies. For instance, one important area of difference was on confounding variables controlled in regression model. The marital status of a woman at giving birth was not included in our model. Similarly, we did not include smoking status during pregnancy and parity in our model as these variables do not meet the criteria to be confounding factors. The choice of confounding factor has an undeniably huge influence on the SES-LBW association and this might explain why our findings deviated from what available literature reported.

The study has a few limitations. Wealth index was computed based on the current (at the time the survey was conducted) household possessions and durable materials, but mothers were asked to provide information on past histories, i.e., LBW incidence over the past 5 years prior to the survey. Over the 5 years period, however, household wealth level may have changed and conclusions drawn might likewise be biased. We also did not differentiate the mechanisms that lead to LBW and to intra uterine growth restriction owing to absence of data on gestational age in the EDHS.

Our observed findings stem from cross sectional study where we cannot figure out the cause-effect relationship between education and LBW. Since our analyses were confined to 14% of the birth sample in the survey, findings should be interpreted with this limitation in mind. However, although the sample we base our analysis on was small compared with the total live birth observations in the DHS data, the sample is not necessarily too small to be used for research. In a resource limited countries like Ethiopia, DHS is the main source of birth weight information, where weight is either not properly recorded in the child’s medical card or mothers may not recall it. This results in small samples with complete birth weight information. More than half of the birth weight sample analyzed came from the richest wealth quintile; the greater representation of participants from well to do family might have affected our findings. However, women were invited to provide response on birth weight if they recalled their babies’ birth weight, and their participation was not dependent on whether they were poor or rich. Therefore, it is likely to be by chance that most women in our analysis were from the richest subgroup. Finally, accuracy of birth weight reports based on mother’s estimate is contingent on her recall capability, and the findings presented in this paper should be taken with caution. However, only those birth weight responses the mother recalled were included in the analysis and the risk that mothers could report on birth weight that they did not remember could be minimal.

### Policy implications and areas of future study

Important policy and research relevant findings stem from the study. Child health interventions in Ethiopia need to take account of the appearance of maternal education gradient in LBW to effectively combat the problem. Since the probability of giving birth to LBW neonate occur among illiterate, primary and secondary complete women differently in decreasing order, interventions need to be designed with this disparity in mind in order to substantially reduce the national burden of the problem. That means, women who are ‘illiterate’ need to be policy-targeted the most, followed by ‘primary’ and ‘secondary’ complete mothers. The existence of a statistically significant maternal educational gradient in LBW helps to propose the idea that a targeted approach would be an effective policy choice to maximize gains against LBW; with the ‘whole population’ approach still functioning to disseminate interventions to the entire female population. However, the fact that our findings being drawn from cross sectional survey may affect usefulness for practice and users of the information contained in the paper and therefore the findings should be used along with other sources on this same topic. We highlight that our evidence was drawn from a relatively small samples and the small sample effect might be there. Finally, our findings would be benefited much from future studies to unpack socioeconomic gradient in LBW using data collected objectively.

## Data Availability

The data that support the findings of this study are available from MEASURE DHS at https://www.dhsprogram.com/data/

## Abbreviations

LBW: Low Birth Weight
DHS: Demographic and Health Survey
DSS: Demographic Surveillance System
EDHS: Ethiopia Demographic and Health Survey
SDG: Sustainable Development Goal
SES: Socioeconomic Status
SSA: Sub-Saharan Africa
WHO: World Health Organization
